# G-Protein-Coupled Receptor 35 Mediates Human Saphenous Vein Vascular Smooth Muscle Cell Migration and Endothelial Cell Proliferation

**DOI:** 10.1159/000444754

**Published:** 2016-04-12

**Authors:** Jennifer E. McCallum, Amanda E. Mackenzie, Nina Divorty, Carolyn Clarke, Christian Delles, Graeme Milligan, Stuart A. Nicklin

**Affiliations:** ^a^Institute of Cardiovascular and Medical Sciences, University of Glasgow, Glasgow, UK; ^b^Molecular Pharmacology Group, Institute of Molecular, Cell and Systems Biology, College of Medical, Veterinary and Life Sciences, University of Glasgow, Glasgow, UK

**Keywords:** G-protein-coupled receptor, Vascular smooth muscle cells, Rho kinase

## Abstract

Vascular smooth muscle cell (VSMC) migration and proliferation is central to neointima formation in vein graft failure following coronary artery bypass. However, there are currently no pharmacological interventions that prevent vein graft failure through intimal occlusion. It is hence a therapeutic target. Here, we investigated the contribution of GPR35 to human VSMC and endothelial cell (EC) migration, using a scratch-wound assay, and also the contribution to proliferation, using MTS and BrdU assays, in in vitro models using recently characterized human GPR35 ortholog-selective small-molecule agonists and antagonists. Real-time PCR studies showed GPR35 to be robustly expressed in human VSMCs and ECs. Stimulation of GPR35, with either the human-selective agonist pamoic acid or the reference agonist zaprinast, promoted VSMC migration in the scratch-wound assay. These effects were blocked by coincubation with either of the human GPR35-specific antagonists, CID-2745687 or ML-145. These GPR35-mediated effects were produced by inducing alterations in the actin cytoskeleton via the Rho A/Rho kinase signaling axis. Additionally, the agonist ligands stimulated a proliferative response in ECs. These studies highlight the potential that small molecules that stimulate or block GPR35 activity can modulate vascular proliferation and migration. These data propose GPR35 as a translational therapeutic target in vascular remodeling.

## Introduction

The use of autologous human saphenous vein (HSV) is the most common surgical intervention for the bypass of occluded multivessel coronary artery disease (CAD), despite the use of alternative sources of graft such as the internal mammary artery. Although successful in reducing overall rates of mortality and morbidity in CAD patients, 50% of long-term vein grafts become occluded due to intimal hyperplasia [1]. The pathophysiology of vein graft disease is defined by a cascade of events leading to neointimal formation and graft occlusion. Migration and proliferation of smooth muscle cells (SMCs) is central to neointimal development, which, if unchecked, leads to occlusion of the graft via superimposed atheroma. Current therapeutic options are limited to repeat intervention to replace the occluded grafts. Vascular remodeling is, therefore, an important target for the development of new therapies.

G-protein-coupled receptors (GPCRs) remain the best-studied class of cell surface receptors and the most tractable family of proteins for the discovery of novel, small-molecule drugs [2,3]. However, even in examples in which tissue distribution and genetic ablation data suggest important roles in pathophysiology, a considerable number of GPCRs remain poorly characterized, and, in a significant number of these cases, the endogenous ligand(s) that activate them remain undefined or of questionable physiological relevance [4,5]. GPR35 is an example of this. Despite initially being discovered over a decade ago as an intronless, open-reading frame on chromosome 2 corresponding to 309 amino acids [6] and kynurenic acid (KNU) being identified as an endogenously generated agonist of the receptor [7], GPR35 remains defined as an orphan, due to questions of the effectiveness of this ligand at the human receptor ortholog [8]. Evidence suggests that plasma KNU is only in the nanomolar range physiologically [9,10], although it has been proposed that levels may reach the micromolar range in inflammation and this may therefore activate GPR35 [7]. However, to date, the consensus would suggest that KNU levels are likely to be insufficient for it to be the true endogenous ligand for GPR35 [11]. Pharmacological characterization of GPR35 has been particularly challenging, both because the nature of the endogenous ligand(s) remains uncertain and because many identified synthetic ligands display substantial selectivity between species orthologs [8,12]. This has greatly restricted the capacity to interrogate the contribution of GPR35 in rodent models of disease, including vascular disease. Furthermore, GPR35 displays a limited capacity to signal via the commonly studied G-protein-mediated pathways. We have previously reported that GPR35 interacts selectively with Gα_13_ [13,14], a G protein often considered to regulate the Rho A/Rho kinase cascade and hence to promote alterations of the cytoskeleton that result in cellular shape change and altered cellular migration [15,16,17]. Other study groups have also suggested that GPR35 can couple to the G_i_/G_o_ group of inhibitory G proteins, leading to ERK1/2 activation, at least in certain cell types [18,19,20]. Moreover, although the identification of novel synthetic agonists of GPR35 has often relied on the ability of agonist-occupied GPR35 to recruit β-arrestin-2 [18,21], the functional outputs and consequences of this interaction remain unclear in terms of cellular regulation.

As well as a number of synthetic agonists [22,23], antagonists from 2 distinct chemical classes have recently been identified. The prototypic exemplars of these are CID-2745687 and ML-145 [24,25]. However, although not appreciated immediately [18], both of these ligands have been shown to be highly selective for the human ortholog of GPR35 [14] and show no significant affinity at rodent orthologs. Thus, although not suitable to help define the role of GPR35 in rodent models, they do potentially provide key pharmacological tools to explore the contribution of GPR35 in human cells and tissues.

Expression of GPR35 is high in the small intestine and also in specific immune cells such as peripheral leukocytes, monocytes and neutrophils [7]. However, there are also reports of expression in other tissues and cell types including in the kidney and heart and in regions of the nervous system linked to pain perception [12]. Furthermore, a second isoform of human (h)GPR35 with an N-terminal extension has been identified, although it displays highly similar pharmacology as the short isoform [18,26]. Although not widely investigated in the cardiovascular system to date, GPR35 has previously been implicated in cardiovascular disease. First, a nonsynonymous, single-nucleotide polymorphism has been reported to have a significant association with CAD in a sibship-based case control cohort [27]. Second, in a microarray screen of heart failure patients, GPR35 was reported to have increased expression levels in failing myocardium and in vitro overexpression of GPR35 in cardiomyocytes induced hypertrophy and decreased cell viability [28]. Moreover, GPR35 knockout mice are reported to have significantly increased blood pressure compared to their wild-type littermates [28]. Most recently, GPR35 expression was reported to be induced by hypoxia-inducible factor-1 in hypoxic cardiomyocytes, leading to changes in the actin cytoskeleton [29].

Here, we have used the developing knowledge of GPR35 pharmacology to explore a role for GPR35 in human primary vascular endothelial cells (ECs) and SMCs. We demonstrate that activation of GPR35 can contribute to the increased migration and proliferation of primary HSV vascular cells and that antagonism of its activity blocks these effects. These data suggest that GPR35 antagonism may be an important, novel and translational therapeutic target.

## Materials and Methods

Materials for the cell culture were from Sigma-Aldrich (Gillingham, Dorset, UK), Life Technologies (Paisley, Strathclyde, UK) or PAA Laboratories Ltd. (Yeovil, Somerset, UK). Polyethylenimine (PEI) linear MW-25000 was from Polysciences Inc. (Warrington, Pa., USA). CID-2745687, ML-145 andzaprinast were purchased from Tocris Bioscience (Bristol, UK).

### Bioluminescence Resonance Energy Transfer

HEK293T cells were maintained in Dulbecco's modified Eagle's medium (DMEM) supplemented with 0.292 g/l L-glutamine and 10% (v/v) newborn-calf serum at 37°C in a 5% CO_2_ humidified atmosphere. Cells were transfected with FLAG-tagged hGPR35 enhanced yellow fluorescent protein (FLAG-hGPR35-eYFP) and β-arrestin-2-*Renilla* luciferase 6 (ratio 4:1), using 1 mg/ml PEI. After 24 h, cells were washed with Hanks' balanced salt solution (pH 7.4), and coelentrazine-h (Promega) was added to a final concentration of 5 μM. Cells were incubated in darkness for 10 min at 37°C before the addition of receptor ligands. Cells were incubated for a further 5 min at 37°C before BRET measurements were performed using a PHERAstar FS reader (BMG-Labtech, Offenburg, Germany). The BRET ratio was calculated as a wavelength emission at 530/485 nm and expressed as the percentage of maximal signal for each ligand [13,14].

### Inositol Phosphate Generation Assays

Inositol phosphate (IP) accumulation was measured using a homogenous time-resolved FRET (HTRF) assay (HTRF IP-One Tb kit, Cisbio Bioassays, Codolet, France). HEK293T cells were transiently cotransfected with FLAG-hGPR35-eYFP and the G-protein chimaera Gα_q/13_5 (a form of Gα_q_ in which the C-terminal 5 amino acids were replaced with the corresponding pentapeptide from Gα_13_) using PEI. After 16-24 h of incubation at 37°C in a 5% CO_2_ humidified atmosphere, the cells were resuspended in IP-One stimulation buffer (10 mM HEPES, 1 mM CaCl_2_, 0.5 mM MgCl_2_, 4.2 mM KCl, 146 mM NaCl, 5.5 mM glucose and 50 mM LiCl, pH7.4) and seeded at 10,000 cells/well in white, solid-bottom, 384-well plates. Ligands were diluted in IP-One stimulation buffer according to the manufacturer's instructions. Antagonist compounds were preincubated with cells for 15 min at 37°C prior to the addition of the agonist. Cells were incubated with ligand(s) for 2 h at 37°C, before the addition of d_2_-conjugated IP monophosphate (IP_1_; 3 μl/well) and anti-IP_1_ Lumi4™-Tb cryptate (3 μl/well) diluted in lysis buffer. After incubation at room temperature for 1 h, HTRF was measured using a PHERAstar FS plate reader (BMG-Labtech). IP_1_ accumulation was measured by the fluorescence ratio of 665 nm/620 nm.

### Quantifying GPR35 Expression

In order to quantify GPR35 expression levels in individual organs, a commercial cDNA panel (Life Technologies) prepared from normal human tissue was utilized. For vascular cells, RNA was extracted from cells plated in 6-well plates using an RNeasy RNA extraction kit as per the manufacturer's instructions (Qiagen, Crawley, UK). Reverse-transcriptase reactions were carried out using a Taqman Multiscribe RT kit with random hexamers according to the manufacturer's instructions. mRNA expression of hGPR35 and ribosomal 18S were quantified by real-time PCR using Taqman chemistries (Applied Biosystems, Warrington, UK). The mRNA expression level of GPR35 in tissues was expressed as a relative quantification (RQ) or ΔCT value normalized to the housekeeper gene ribosomal 18S, and was further normalized to levels in the heart. For quantification of expression in cells, the GPR35 copy number per nanogram of total RNA was calculated by constructing a standard curve for FLAG-hGPR35-eYFP in pcDNA3 (7046 bp) [21]. The mass per copy was calculated using the formula m = (n)(1/Avogadro's number)(average molecular weight of 1 bp), where n = plasmid bp. Serial dilutions of 30-300,000 copies were added per TaqMan reaction.

### Isolation and Culture of Primary Human Vascular ECs and SMCs

Vascular cells were grown from medial explants from HSV segments obtained from male and female patients undergoing coronary artery bypass grafting and who gave their informed consent. Ethical permission was obtained from the West of Scotland Research Ethics Committee 4 (reference No. 10/S0704/60) and the investigation conformed to the principles outlined in the Declaration of Helsinki. HSV SMCs were maintained in DMEM with 4,500 mg/l glucose supplemented with 15% (v/v) fetal-calf serum (FCS) and 100 IU/ml penicillin, 100 IU/ml streptomycin and 2 mmol/l L-glutamine. HSV ECs were maintained in EC complete medium (TCS Cellworks, UK) and supplemented with 20% FCS (PAA Laboratories, Yeovil, UK).

### Cellular Morphology Assays

HSV SMCs or ECs were seeded in 6-well plates at 1 × 10^5^ cells/well and quiesced for 48 or 24 h, respectively. Following 45 min of exposure to GPR35 ligands at 37°C, the cells were fixed using 4% paraformaldehyde, and stained with TRITC F-actin phalloidin at 5 μg/ml for 1 h at room temperature (Sigma). Cells were washed and mounted with Prolong® Gold Antifade reagent with DAPI (Invitrogen) and were then imaged using a spinning disk-structured illumination Viva Tome device and analyzed using ImageJ software. Equivalent studies were performed on the Flp-In™ T-REx™ 293 cell line harboring FLAG-hGPR35-eYFP at the Flp-In T-REx locus [14,21]. In such cells, expression of the protein located at the Flp-In T-REx locus is achieved only upon addition of doxycycline or tetracycline. In the absence of treatment with doxycycline or tetracycline, such cells act as a negative control.

### Scratch-Wound Healing Migration Assay

Cells were seeded in 6-well plates at 3 × 10^5^ cells/well, grown to confluence and then quiesced for 48 or 24 h for VSMC and EC, respectively. Three horizontal scratches per well were created before stimulation with various concentrations of pamoic acid (Sigma-Aldrich) or zaprinast in serum-free DMEM. In a number of experiments, a GPR35 agonist was coadded with 100 nM of either of the human-specific GPR35 antagonists, CID-2745687 and ML-145. In equivalent studies, the structurally and mechanistically distinct Rho A pathway inhibitors, Y-27632 [30,31,32] or Y-16 [33], were coadministered with a GPR35 agonist. Images of the cells were acquired at time zero, followed by incubation at 37°C. The cells were imaged up to a maximum period of 36 h. Percentage migration was assessed by measuring the distance (μm) between cells migrating into the wound area in 10 random fields of view per scratch over the time course using ImageJ software.

### Cell Proliferation

HSV SMCs and ECs were seeded in clear, 96-well plates at a density of 5 × 10^3^ cells/well and quiesced in full medium, without serum for 48 or 24 h, respectively. The cells were then stimulated with a range of concentrations of ligands ±5% FCS, over a period of 48 h (SMCs) or 24 h (ECs). Cell proliferation was assessed using either Cell Titer 96 aqueous nonradioactive cell proliferation assay (MTS; Promega, Madison, Wis., USA) or the 5-bromo-2′-deoxyuridine (BrdU) cell proliferation assay kit (Cell Signaling Technology, UK) according to manufacturer's instructions. Colorimetric output was measured on a Wallac VICTOR^2^ plate reader at absorbance values of 490 or 450 nm for the MTS and BrdU assays, respectively.

### Statistical Analysis

All data are provided as mean ± SEM and were analyzed using GraphPad Prism® software. Student's t test for paired data and one-way ANOVA with Dunnett's test for multiple comparisons were applied and a statistical difference was considered if p < 0.05. Taqman, β-arrestin-2 and IP_1_ accumulation experiments were performed in duplicate. All other experiments were performed in triplicate on each occasion. N values for each experiment in the figure legends indicate the number of times an experiment was repeated. Individual cell numbers/numbers of measurements made for migration and cell size experiments are indicated separately.

## Results

### Pharmacological Assessment of GPR35 Ligands at hGPR35

**Fig. 1:**
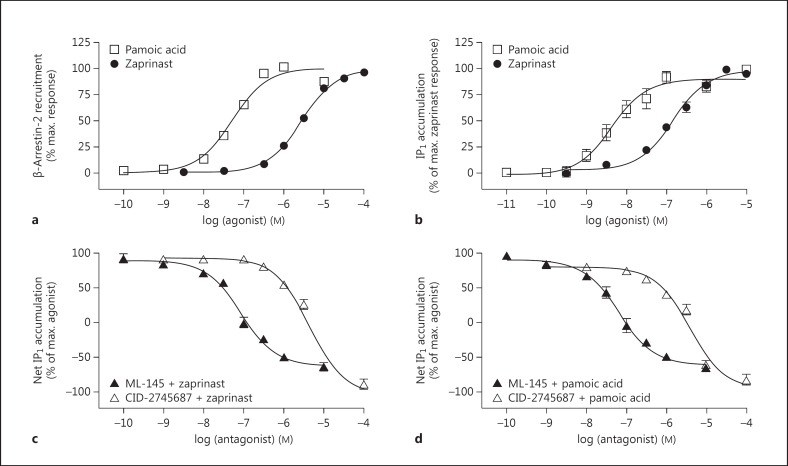
The GPR35 antagonists CID-2745687 and ML-145 blocked agonist-induced hGPR35 activation in 2 distinct assays. Activation of hGPR35 by varying concentrations of the agonists pamoic acid and zaprinast in a β-arrestin-2 interaction assay (**a**), where FLAG-hGPR35-eYFP was cotransfected with β-arrestin-2-*Renilla* luciferase in HEK293T cells (n = 6), or an IP_1_ accumulation assay (**b**), where FLAG-hGPR35-eYFP was cotransfected with Gα_q/13_5 in HEK293T cells (n = 5). hGPR35 activation following cotransfection of FLAG-hGPR35-eYFP and Gα_q/13_5 in HEK293T cells measured via an IP_1_ accumulation assay was inhibited in a concentration-dependent manner following exposure to increasing concentrations of ML-145 and CID-2745687 in the presence of either zaprinast (**c**; n = 5) or pamoic acid (**d**; n = 7) at EC_80_ concentrations of the agonist ligand. Data are shown as means ± SEM.

Zaprinast and pamoic acid have both been reported to be agonist ligands of hGPR35 [21]. This was confirmed initially by measuring β-arrestin-2 recruitment to the receptor using BRET studies. These were performed in HEK293T cells transfected to transiently coexpress a form of hGPR35 C-terminally tagged with eYFP and β-arrestin-2 tagged with *Renilla* luciferase (fig. 1a). As shown previously [21], pamoic acid (pEC_50_ = 7.30 ± 0.06) was more potent than zaprinast (pEC_50_ = 5.44 ± 0.01; means ± SEM, n = 6). Both these ligands were also able to promote the production of IPs when hGPR35 was transiently coexpressed with the chimeric G protein Gα_q/13_5 [34]. In this assay, which reflects the capacity of the receptor to interact with the Rho A-linked G protein Gα_13_ [17,34], both zaprinast (pEC_50_ = 6.83 ± 0.06) and pamoic acid (pEC_50_ = 8.36 ± 0.11; means ± SEM; n = 5 in each case) were more potent than recorded in the β-arrestin-2 recruitment assay (fig. 1b). This reflects that there is frequently receptor reserve in G-protein-mediated signal transduction events that is not routinely evident in the β-arrestin-2 recruitment assay. Using this IP accumulation assay as a surrogate measure of interactions of the receptor with Gα_13_, both ML-145 and CID-2745687 were able to fully block the agonist effect of either zaprinast (fig. 1c) or pamoic acid (fig. 1d) at hGPR35, confirming their suitability to act as highly selective inhibitors of GPR35 in cells and tissues of human origin. Once again, as described previously [14], ML-145 displayed some 50-fold higher affinity than CID-2745687 as an hGPR35 antagonist (fig. 1c, d). As noted previously, GPR35 displays a level of constitutive activity in G-protein-mediated assays, and both CID-2745687 and ML-145 acted as inverse agonists [14], able to suppress constitutive activity as well as the agonist effects of pamoic acid and zaprinast in the IP_1_ accumulation assay (fig. 1c, d).

### Expression of GPR35 in Human Vascular Cells and Tissue

**Fig. 2:**
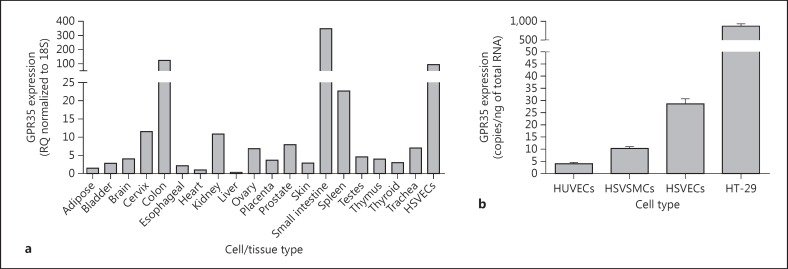
GPR35 is expressed in human VSMCs and ECs. hGPR35 mRNA expression in human tissues and HSV ECs (n = 3) normalized to 18S and expressed as RQ relative to hGPR35 expression in the heart (**a**) or in a range of immortalized and primary cells (**b**) was quantified via TaqMan. HSV ECs (n = 3), and SMCs (n = 3), HUVECs (n = 3) and HT-29 colon carcinoma cells are expressed as copy numbers per nanogram of RNA. Data are shown as means ± SEM.

Next, expression of GPR35 was investigated at the mRNA level using a human tissue cDNA panel (fig. 2a). Consistent with previous reports [7,35], high levels of hGPR35 were detected in the small intestine, colon and spleen. It was also noted that hGPR35 mRNA expression could be detected in many other tissues of the body including the kidney and cervix, albeit at lower levels (fig. 2a). Interestingly, expression was also high in HSV ECs (fig. 2a). To explore the vascular expression of GPR35 more extensively, RNA from HSV ECs, HSV SMCs and human umbilical-vein ECs (HUVECs) was reverse-transcribed and examined via real-time qRT-PCR and copy number in each cell type calculated (fig. 2b). Substantial levels of GPR35 expression were detected in all 3 vascular cell types with copy numbers of 3.99 ± 0.61 copies/ng total RNA in HUVECs, 28.5 ± 2.2 copies/ng total RNA in HSV ECs and 10.2 ± 0.76 copies/ng total RNA in HSV SMCs, although they were significantly lower than the levels detected in the colon carcinoma cell line, HT-29 (871.0 ± 63.47 copies/ng total RNA; fig. 2b). Interestingly, previous studies have suggested levels of expression of GPR35 in HUVECs to be barely detectable [36].

### Effects of GPR35 Stimulation on the HSV SMC Cytoskeleton

**Fig. 3:**
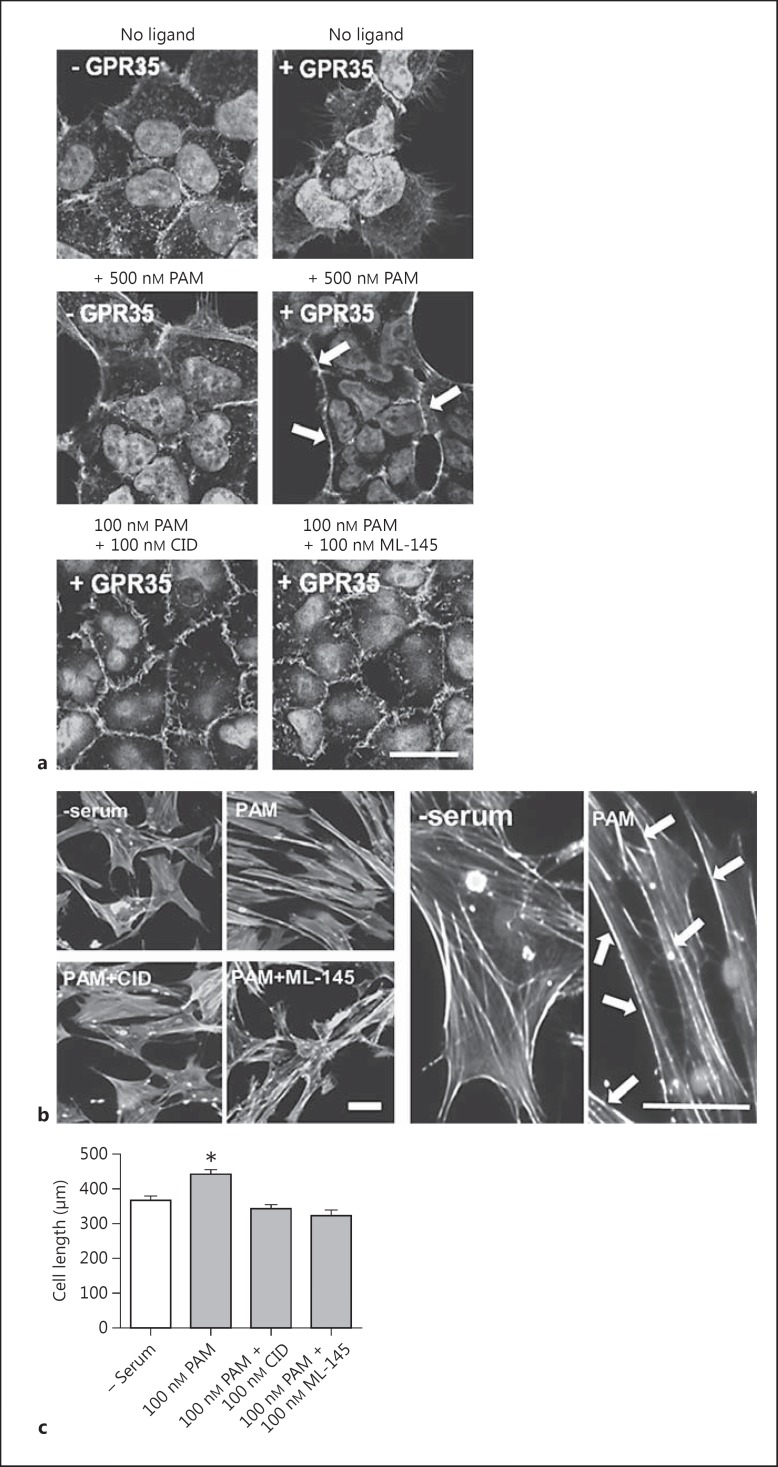
Pamoic acid induces changes in cell morphology which are blocked by the hGPR35-selective antagonists CID-2745687 and ML-145. **a** Images of doxycycline-inducible Flp-In T-REx 293 cells stably expressing FLAG-hGPR35-eYFP ± 100 ng/ml doxycycline (±GPR35) stimulated with 500 nM pamoic acid (PAM) 24 h later, either alone or in the presence of 100 nM CID-2745687 (CID) or 100 nM ML-145 for 45 min (n = 4 per condition). Cells were stained for F-actin using TRITC actin phalloidin and imaged via spinning-disk illumination VivaTome™ microscopy. Scale bar = 20 µm. **b** Representative images of quiescent HSV SMCs fixed and stained for F-actin following stimulation with 100 nM of PAM ± antagonists CID or ML-145 for 45 min (n = 3). Scale bars = 50 µm. Arrows highlight changes in actin filament organization following pamoic acid stimulation. **c** Quantification of HSV SMC length (µm) in the presence of 100 nM PAM ± antagonists CID and ML-145 (3 experiments; 40-60 cells measured/condition/experiment). Data are shown as means ± SEM. * p < 0.001 versus serum control, analyzed via 1-way ANOVA with Dunnett's test for multiple comparisons.

To date, there have been no direct studies investigating the functional outcomes of GPR35 activation in human vascular cells, although the capacity of intercellular adhesion molecule (ICAM)-1 expressing HUVECs to interact with leukocytes in a GPR35-dependent manner has been reported [36]. Since stimulation of GPR35 is known to result in the activation of G_α13_ (fig. 1b) [13], and this is frequently associated with downstream signaling via Rho A [17], the effects of GPR35 ligand exposure on HSV SMC cytoskeleton arrangement and migration were examined (fig. 3). Prior to this, the effect of GPR35 activation in Flp-In T-REx 293 cells harboring FLAG-hGPR35-eYFP at the Flp-In T-REx locus [21] was analyzed. In the absence of doxycycline induction, FLAG-hGPR35-eYFP is not expressed by these cells, providing a negative control for possible nonreceptor-mediated effects of the ligands. Here, exposure to pamoic acid (500 nM) did not induce any changes in cell morphology (fig. 3a). Moreover, in the absence of ligand addition, induction of expression of FLAG hGPR35-eYFP also did not produce alteration in cell morphology. However, in cells treated with doxycycline to induce FLAG-hGPR35-eYFP expression, exposure to pamoic acid for 45 min did now produce alterations in cell morphology, with cells becoming contracted in appearance and alteration of the actin cytoskeleton, from its resting disorganized arrangement, to defined structures localized at the plasma membrane (fig. 3a). These effects were blocked when pamoic acid was coincubated along with either of the GPR35 antagonists CID-2745687 or ML-145 (fig. 3a), confirming that these alterations in cellular morphology reflected the activation of GPR35. Next, the effects of the ligands on HSV SMC morphology and cytoskeletal rearrangement were examined (fig. 3b, c). Incubation with the hGPR35-selective agonist pamoic acid resulted in changes in the HSV SMCs, in which the resting unorganized actin cytoskeleton rearranged into aligned stress fibers at the plasma membrane, with cells becoming elongated in appearance in comparison to agonist-free conditions (fig. 3b). These effects were lacking when cells were coincubated with pamoic acid and either CID-2745687 or ML-145 (fig. 3b). Quantification of cell length using ImageJ revealed that addition of the hGPR35 agonist pamoic acid induced a significant increase in length of the HSV SMCs from 366.2 ± 12.5 to 440.6 ± 15.7 μm (p < 0.001; fig. 3c). This effect was completely abolished following coincubation of pamoic acid with either CID-2745687 or ML-145, suggesting that in primary human vascular cells, the specific activation of GPR35 was able to induce changes in cell architecture, leading to changes in cell size (fig. 3c).

### Effects of GPR35 Stimulation on HSV SMC Migration and Proliferation

**Fig. 4:**
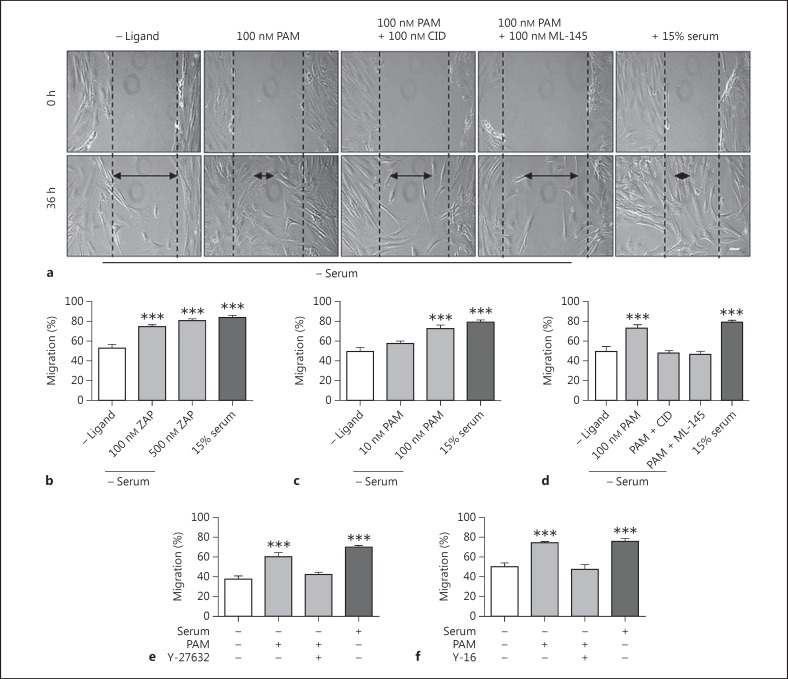
GPR35 mediates HSV SMC migration via the Rho A-ROCK signaling axis. **a** Representative images of migration in confluent, quiescent HSV SMCs in a scratch-wound healing assay, following exposure to 100 nM pamoic acid (PAM) ± 100 nM of the GPR35 antagonists CID-2745687 (CID) or ML-145. Scale bar = 50 µm. HSV SMC migration following exposure to the agonists zaprinast (ZAP; n = 4; **b**), PAM (n = 8; **c**) or PAM ± the GPR35 antagonists CID or ML-145 (n = 5; **d**) was quantified at 0 and 36 h (n = 4- 8; 90 individual measurements/condition/experiment). Scratch width was measured via ImageJ and expressed as percent migration between 0 and 36 h. **e**, **f** Migration of confluent, quiescent HSV SMCs in the presence of the hGPR35 agonist PAM ± the Rho A pathway inhibitors Y-27632 (**e**) or Y-16 (**f**), measured by scratch-wound healing assay (n = 5; 90 individual scratch measurements/condition/experiment). Data are shown as means ± SEM. *** p < 0.001 versus serum control, analyzed via 1-way ANOVA with Dunnett's test for multiple comparisons.

The role of GPR35 in the migration of HSV SMCs was assessed using the GPR35 agonists zaprinast and pamoic acid in a scratch-wound healing assay. Both GPR35 agonists significantly enhanced HSV SMC migration with 500 nM zaprinast inducing migration by 28.2 ± 1.8% and 100 nM pamoic acid by 23.9 ± 3.3% higher than that observed in comparison to serum-free control-quiesced HSV SMCs (p < 0.001; fig. 4a-c). Moreover, at the most effective concentrations, the GPR35 agonists promoted migration as successfully as FCS (30.1 ± 2.3%). Next, the effects of coincubating cells with 100 nM pamoic acid and 100 nM of either of the hGPR35 antagonists, CID-2745687 and ML-145, was studied. Both antagonists completely blocked pamoic-acid-induced migration (fig. 4a, d). Neither antagonist had any effect on basal HSV SMC migration when used in the absence of agonist (data not shown).

**Fig. 5:**
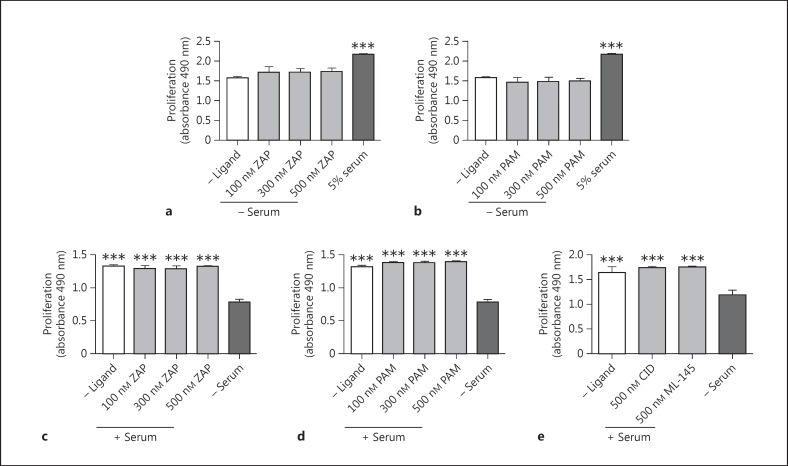
Modulation of GPR35 activation does not stimulate HSV SMC proliferation. The proliferation of quiescent HSV SMCs in the presence of increasing concentrations of zaprinast (ZAP; n = 3; **a**) or pamoic acid (PAM; n = 3; **b**) was measured by MTS assay at 48 h and expressed as A.U. The ability of ZAP (n = 4; **c**) or PAM (n = 4; **d**) to block HSV SMC proliferation stimulated by exposure to 5% FCS was quantified using an MTS assay. **e** Assessment of the effects of the GPR35 antagonists CID-2745687 (CID; n = 3) or ML-145 (n = 3) on the serum-induced proliferation of HSV SMCs was measured via MTS assay at 48 h. Data are shown as means ± SEM. *** p < 0.001 versus serum control, analyzed via 1-way ANOVA, with Dunnett's test for multiple comparisons.

Previous publications have reported effective and selective coupling of GPR35 to Gα_13_[21], and it is established that Gα_13_ can promote Rho A activation and signaling via the Rho kinases (ROCK) 1/2 and their effectors [37]. Therefore, to assess the involvement of this signaling pathway in GPR35-promoted HSV SMC migration, pamoic acid was coincubated with either of 2 mechanistically distinct Rho A pathway inhibitors, Y-27632 (10 μM) [32] and Y16 (10 μM) [33] (fig. 4e, f). Both these inhibitors prevented pamoic-acid-induced migration (p < 0.001), suggesting that GPR35 activation mediates these cell-migratory effects via the Rho A-ROCK 1/2 signaling axis. Next, HSV SMCs were exposed to GPR35 ligands and cell proliferation was quantified. Across a range of concentrations, neither zaprinast nor pamoic acid produced a proliferative response in unstimulated, quiesced HSV SMCs (fig. 5a, b). Moreover, neither zaprinast nor pamoic acid altered proliferation when HSV SMC proliferation was stimulated by the exposure of quiesced HSV SMCs to FCS (fig. 5c, d). This was also the case for the hGPR35 antagonists CID-2745687 and ML-145 (fig. 5e), indicating that the effect of serum on HSV SMC proliferation does not reflect the presence of GPR35 agonists within serum.

### Effects of GPR35 Stimulation on HSV EC Migration and Proliferation

**Fig. 6:**
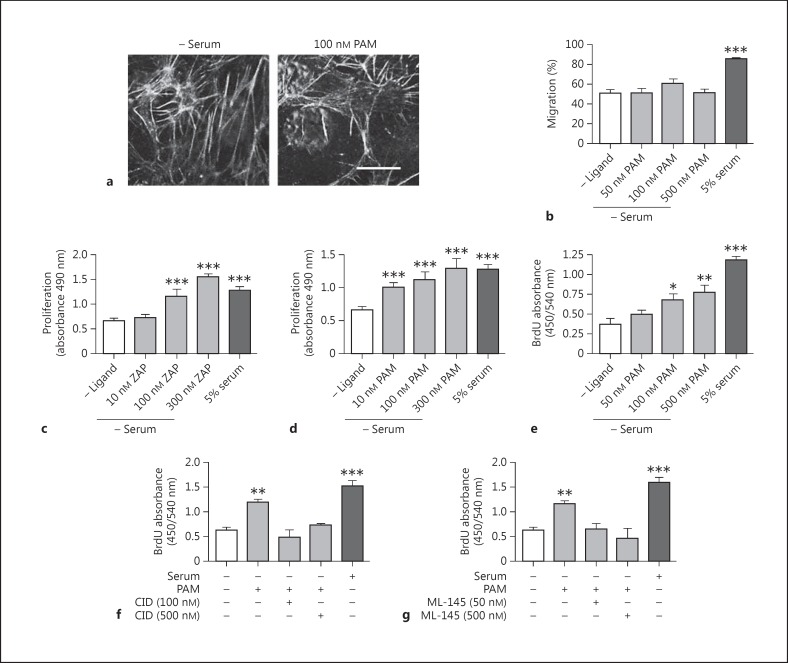
GPR35 agonists stimulate HSV EC proliferation. **a** Representative images of the HSV EC cytoskeleton, stained for F-actin following stimulation with 100 nM pamoic acid (PAM) for 45 min (n = 3). Scale bar = 50 µm. **b** Migration of quiescent HSV ECs is unaffected in the presence of increasing concentrations of PAM (50, 100 and 500 nM; n = 2; 90 individual scratch measurements/condition/experiment), measured by scratch-wound healing assay. Increasing concentrations of zaprinast (ZAP; n = 3; **c**) or PAM (n = 3; **d**) induced proliferation in quiescent HSV EC, as measured by MTS or BrdU assay (**e**) following 24 h of ligand exposure. HSV EC proliferation in response to GPR35 agonists was blocked via coincubation with increasing concentrations (50-500 nM) of the GPR35 antagonists CID-2745687 (CID; **f**) or ML-145 (**g**), measured via BrdU assay 24 h after stimulation (n = 2 per agonist/antagonist pair). Data are shown as means ± SEM. * p < 0.05, ** p < 0.01, *** p < 0.001 versus serum control, analyzed via 1-way ANOVA with Dunnett's test for multiple comparisons.

Impaired EC regeneration and function is implicated in the incidence of vein graft occlusion, and so the effects of GPR35 ligands on HSV EC movement and proliferation were assessed next. Exposure of HSV ECs to pamoic acid did not induce any alterations in morphology or cytoskeletal architecture (fig. 6a). Furthermore, using a scratch-wound healing assay, no effect on HSV EC migration was observed whereas the addition of 5% serum produced a marked increase in cell migration (fig. 6b). Next, proliferation was quantified. In contrast to what was observed in HSV SMCs, the exposure of quiesced ECs to increasing concentrations of zaprinast or pamoic acid induced concentration-dependent increases in proliferation [control 0.67 ± 0.05 arbitrary units (A.U.); 300 nM zaprinast 1.55 ± 0.12 A.U.; 300 nM pamoic acid 1.23 ± 0.15 A.U.], comparable in extent to that achieved with FCS (1.29 ± 0.06 A.U.; fig. 6c, d). This effect of pamoic acid was replicated in an independent assay by assessing BrdU incorporation in HSV ECs under identical conditions (fig. 6e-g), although the extent of the effect of pamoic acid in this assay was less than that produced by the addition of serum (fig. 6e-g). The proliferation induced by concentrations of pamoic acid up to 500 nM was completely abolished (p < 0.001) in the presence of either CID-2745687 or ML-145 (fig. 6f, g). These data suggest that GPR35 activation can mediate HSV EC proliferation and that the role of this receptor in migration and proliferation may be cell type-dependent.

## Discussion

This study is the first to investigate the role GPR35 may play in primary human VSMC and EC proliferation and migration. Recent literature has begun to highlight a potential role for GPR35 in a number of disease pathologies including hypertension and heart failure [28,29], asthma [38], pain [18,39], inflammatory bowel disease [40] and both ulcerative colitis and primary sclerosing cholangitis [41]. To date, GPR35 has been a challenging receptor to investigate because of uncertainty about the endogenous ligand(s) that activate it [8,22] and because the majority of synthetic GPR35 agonist ligands display either marked species ortholog selectivity or are known to have molecular targets in addition to GPR35 [22]. For example, the tryptophan metabolite KNU was the first reported endogenous GPR35 ligand [7], and although subsequently confirmed at rat GPR35 with micromolar potency, the activation of hGPR35 was almost undetectable in response to this ligand [21,42]. Considering that circulating plasma concentrations are reported in nanomolar ranges, although these may increase substantially in various inflammatory conditions [36], KNU is an unlikely endogenous ligand at hGPR35 [8]. More recently, the chemokine CXCL17 was reported to be an endogenous ligand at GPR35 [43]. Transfection of GPR35 into a GPR35-negative cell line led to the induction of CXCL17-mediated calcium flux following exposure of the cells to CXCL17, and induced chemotaxis in THP-1 monocyte cells expressing GPR35 [43]. This is an interesting finding which requires further study to be consolidated. In the search for tool compounds able to interrogate the role of GPR35, a large number of ligands with an agonist function but modest potency have been reported [12,22]; the first to be identified and the most widely utilized is zaprinast [44]. Although zaprinast has the advantage of displaying agonism at rodent orthologs of GPR35 as well as at the human receptor with moderate potency and remains the customary ligand of reference in chemical screens targeting novel GPR35 ligands, it is limited in its use by its known potency as an inhibitor of cGMP phosphodiesterase subtypes. Many other recently described GPR35 active ligands also have other targets. Indeed, amlexanox [26] has recently been described as an inhibitor of both IKK-ε and TBK1 [45] and is used to target these kinases in in vivo studies, despite displaying a markedly lower affinity at these kinases than at GPR35 [26,46]. As such, together with zaprinast as a reference ligand, we employed pamoic acid as a GPR35 agonist in these studies. Pamoic acid has a high potency at hGPR35 but very modest effects at the rodent orthologs and can therefore only be used usefully in studies employing human rather than rodent cells and tissues. Perhaps even more importantly from a pharmacological perspective, there are a very limited number of useful antagonists of GPR35. Moreover, the 2 key antagonists ML-145 and CID-2745687 are also entirely specific for the human receptor, displaying no useful affinity at either rat or mouse GPR35 [14]. Furthermore, it has been previously demonstrated that ML-145 displays >1,000-fold selectivity for GPR35 compared to the most closely related GPCR, GPR55 [24], and since both ML-145 and CID-2745687 were utilized in the nanomolar range, it is unlikely that they showed promiscuous activity at other closely related receptors to GPR35, such as GPR55 and GPR23. As such, whilst studies on human cells and tissues are obviously most relevant for translation in a therapeutic context, our study was restricted to human tissue-derived cells. Despite this, the pharmacological characteristics of these antagonists mean that they provide a currently unique opportunity to probe the functional importance of GPR35 in disease-relevant, human cell models.

Using these combinations of agonists and antagonists, we demonstrate a potentially important role for GPR35 in the migration of primary HSV SMCs. These cells express readily detectable levels of GPR35 mRNA that are comparable to those found in the spleen and small intestine. The data demonstrate that coincubating a wounded monolayer of HSV SMCs with one of the hGPR35-specific antagonists, CID-2745687 and ML-145, abolished increased migration induced by either of the GPR35 agonists, zaprinast or pamoic acid, thus supporting a specific role for GPR35. Investigation of the cellular morphology and cytoskeletal rearrangement in HSV SMCs revealed significant architectural reorganization of the actin cytoskeleton and, similarly, these changes were also prevented by the coaddition of the GPR35 antagonists. We hypothesized that the activation of ROCK 1/2 and subsequent myosin light-chain phosphorylation would be directly linked to the reorganization of the actin cytoskeleton, leading to vascular cell contraction and migration [30,47]. To examine this more closely, the effect of the presence of 2 mechanistically distinct Rho A pathway inhibitors, Y-27632 and Y-16, on the GPR35 agonist-induced migration of HSV SMCs was assessed, and both were able to block the effects of GPR35 agonism on migration. This is entirely consistent with previous observations of the selective activation of Gα_13_ by GPR35 agonism [13], a mode of coupling that we have suggested is likely integral to the functional roles of GPR35 [8]. Other study groups have reported similar effects of Rho kinase 1/2 inhibitors in VSMC migration induced by PDGF and lysophosphatidic acid in a collagen matrix migration model [48]. Despite ongoing efforts to target Rho A and downstream mediators in disease settings, blocking this pathway across a broad spectrum of cell types may not be attractive therapeutically due to unwanted, off-target side effects [49,50]. It is proposed, therefore, that compounds which mimic this effect, such as GPR35 antagonists, may have distinct therapeutic advantages with respect to cell and tissue specificity.

By further exploring the role of GPR35 in proliferation, we showed that, at least in HSV SMCs, neither GPR35 activation nor inhibition produced any effects on cell proliferation. Importantly, this suggests that the finding of increased migration is not confounded by increased cell numbers through cell division. Conversely, HSV ECs demonstrated significantly enhanced proliferation in the presence of increasing concentrations of either zaprinast or pamoic acid without effects on migration. Although this might be unexpected, it is well established that GPCRs often exhibit diverse effects amongst different cell populations [51,52]. Ruling out potential issues regarding receptor specificity, it was further demonstrated that the proliferation induced by pamoic acid was blocked by the presence of the antagonists CID-2745687 and ML-145. Taken together with the knowledge that ML-145 has been shown to display >1,000-fold selectivity for GPR35 over its most closely related receptor GPR55, the specificity of the antagonists is likely not an issue here, and, therefore, it can be concluded that GPR35 activation plays a role in mediating proliferation in HSV ECs.

Importantly, this is the first study to report significant expression levels of GPR35 in vascular cells and a functional role for hGPR35 in the setting of vascular remodeling. Future investigations that address the mechanism of the effects of GPR35 on vascular EC proliferation will be integral in our understanding of its true function in the vasculature. However, our findings strongly suggest that antagonists of GPR35 may be of beneficial therapeutic effect with respect to attenuating vascular occlusion in the setting of vein graft failure.

## Disclosure Statement

There were no disclosures.
